# Diabetes specialist nurses' reasoning on their professional practice in contemporary primary diabetes healthcare: a focus group study

**DOI:** 10.1080/17482631.2026.2633554

**Published:** 2026-02-20

**Authors:** Alma Dautovic, Eva Brink, Ulla Fredriksson-Larsson

**Affiliations:** a Department of Health Sciences, University West, Trollhättan, Sweden

**Keywords:** Diabetes mellitus, delivery of health care, person-centred care, professional role, work-integrated learning, qualitative research

## Abstract

**Introduction:**

The prevalence of type 2 diabetes is increasing alarmingly worldwide. Evidence consistently shows that nurse-led care by diabetes specialist nurses is crucial for delivering efficient front-line primary diabetes management. This study aimed to explore diabetes specialist nurses’ experiences of the prerequisites for person-centred care and for their professional practice in primary diabetes healthcare.

**Methods:**

A total of 16 diabetes specialist nurses from western Sweden participated in four focus-group discussions during May 2025. The data were analysed to identify common themes among the participants' experiences according to Krueger and Casey’s descriptions.

**Results:**

The analysis resulted in one main theme: “maintaining professionalism in a changing and strained primary diabetes care”. This theme included five subthemes that described diabetes specialist nurses’ experiences regarding the prerequisites for person-centred care and professional practice. The diabetes specialist nurses reported daily challenges in delivering person-centred care in contemporary primary diabetes healthcare. Their professional commitment was evident in their clinical practice and in their efforts to maintain professional competence. Despite emphasising the importance of long-term, person-centred patient engagement in diabetes care, they face limited time to interact with patients and insufficient managerial support for their professional learning.

**Discussion:**

The findings underscore diabetes specialist nurses’ need for organisational support, particularly from managers who value and actively promote person-centred care, as well as opportunities for continuous professional development. Taken together, such supportive measures could lay the groundwork for a sustainable primary healthcare system for diabetes in the future.

## Introduction

Globally, an estimated 589 million adults aged 20–79 live with diabetes, and, by the year 2050, this number is expected to rise to 852.5 million. Type 2 diabetes mellitus (T2DM) accounts for the vast majority (over 90%) of diabetes cases worldwide. T2DM can be prevented or delayed, and there is increasing evidence that remission of T2DM may be possible with the proper support and intervention in the early stages of the condition (International Diabetes Federation, 2025).

Nursing professionals, according to the World Health Organisation (2020), play a key role in delivering holistic, person-centred care (PCC). They coordinate treatment, administer medications, manage pain, educate patients and their families, supervise staff and support research and health promotion initiatives. These tasks help ensure quality, safety and continuity of care in all healthcare settings.

Professional nursing values were defined in a conceptual analysis as fundamental principles of professional nursing. These include human dignity, integrity, altruism and justice, which guide standards of care, professional conduct and evaluation of their practice (Schmidt & McArthur, 2018). Internationally, professional nursing encompasses several key aspects, including who provides care, their roles and responsibilities, where they work, the significance of their role and the complexity of their work. Licensed registered nurses and advanced-practice registered nurses practise autonomously and take responsibility for health promotion, disease prevention and the delivery of compassionate care across diverse settings and throughout all stages of life (American Nurses Association, 2025). In Sweden, diabetes specialist nurses (DSNs) are registered nurses with advanced training in diabetes care who work in line with international and national standards. Swedish diabetes care is decentralised in such a way that persons with T2DM are enroled at primary healthcare centres and are usually treated by multidisciplinary teams comprising DSNs, physicians and other professionals (National Board of Health and Welfare, 2018).

The foundation of managing T2DM is to improve care by encouraging people living with diabetes to adopt a lifestyle that includes a healthy diet, regular physical activity, quitting smoking and maintaining a healthy body weight, all of which help prevent or delay diabetes complications (International Diabetes Federation, 2025). For these lifestyle changes to be effective, professionals play a vital part in guiding and supporting persons with diabetes. Aldahmashi et al. (2024) found in their integrative review that patient education is a core responsibility of DSNs within multidisciplinary teams. DSNs were recognised for their vital role in delivering front-line primary care and conducting follow-ups with patients with T2DM. Similarly, a narrative review by Zhu et al. (2024) showed the importance of DSNs in healthcare practice: they educate patients on disease management, the prevention of complications and self-care, thereby reducing the burden on healthcare systems and contributing to improving treatment outcomes for persons with diabetes.

The review with synthesis of multiple studies by Lawler et al. (2019) demonstrated that DSNs help reduce hospital lengths of stay, minimise inpatient complications, educate both patients and healthcare professionals and improve overall patient satisfaction. In this vein, a Swedish randomised controlled trial by Jutterström et al. (2016) showed that nurse-led diabetes care improved blood glucose control, as evidenced by reduced glycated haemoglobin levels in patients with T2DM compared to those receiving standard diabetes care.

In alignment with these findings, the Swedish government (Swedish Government Official Reports, 2020) has committed to advancing PCC as a cornerstone in the ongoing transformation toward a modern, equitable healthcare system. PCC recognises each person’s unique capacity to practise self-care as opposed to a general assumption about people’s ability to manage symptoms or disease. Indeed, research has shown that self-care interventions should be implemented to improve and promote patients’ health maintenance (El-Osta, 2019). PCC is an approach to establishing routines that initiate the partnership between patient and caregiver through patient narratives, work the partnership through shared decision-making and safeguard the partnership by documenting the narrative in daily clinical practice (Ekman et al., 2011).

A profession is a career that requires specialised experiences and skills based on theoretical knowledge. Professionals are recognised by society for their authority as compared to nonprofessionals, who lack specific theoretical knowledge or experiences (Greenwood, 1957). The term “nursing” encompasses both the profession and its practice, which is carried out through the work of the members of the nursing profession—the nurses. Nursing is a profession committed to ensuring equitable access to the highest attainable standard of health through collaborative, culturally safe and PCC (White et al., 2025). Given the central role of DSNs in delivering such care, this study aimed to explore DSNs’ experiences of the prerequisites for PCC and for their professional practice in primary diabetes healthcare.

## Materials and methods

### Design

A qualitative research design employing focus-group discussions (FGDs) was used to collect data. Focus-group methodology is grounded in the interaction among participants, which facilitates a collective understanding and insight into how members of a targeted group think and communicate (Ivanoff & Hultberg, 2006; Kitzinger, 1995). When participants with diverse experiences and opinions engage in discussion, new insights can arise at both the individual and collective levels, thereby contributing to both personal and collective learning outcomes (Krueger & Casey, 2015). To ensure compliance with the quality requirements for qualitative research, the consolidated criteria for reporting qualitative studies checklist was used in this study (Tong et al., 2007; see Appendix A).

### Setting and participants

The participants were purposefully recruited from ten different primary healthcare centres in western Sweden in May 2025. The first author contacted the primary healthcare managers by phone to obtain permission to approach DSNs at the healthcare centres to request their voluntary participation in the current study. After obtaining permission, invitations to participate were extended to the DSNs along with information about the study. Subsequently, a new contact was made via phone or email to address any potential questions and to emphasise the voluntary nature of participation. Once participants agreed to take part in the study, a focus group was scheduled.

Initially, there were nineteen DSNs who agreed to participate, but three later declined due to their heavy workloads. A total of sixteen DSNs participated in four FGDs, each of which consisted of three to five participants. The participants were allocated to the groups primarily based on their availability and scheduling preferences. Methodological guidance recommends conducting FGDs until rich, consistent patterns of responses can be clearly identified (Ivanoff & Hultberg, 2006; Krueger & Casey, 2015), which occurred after the fourth FGD in this study.

The homogeneity considering common features and the heterogeneity of differing features among participants were reassessed when the focus groups were formed, which was essential to encompass the diversity within the target group (Ivanoff & Hultberg, 2006; Krueger & Casey, 2015).

### Ethical statement

Ethical approval for this study was obtained from the Swedish Ethical Review Authority (Dnr. 2022-00790-01), an independent body as required by national legislation. Approval was granted to the research team, led by the last author. The study adhered to the Declaration of Helsinki on Medical Research Ethics and Standards (World Medical Association, 2024). Prospective participants were contacted only after their managers at the primary healthcare centres approved the request. Demographic information and informed consent forms were sent to participants and later resent by them via traditional mail. All participants provided written informed consent before the FGDs were conducted.

### Focus groups

The FGDs were conducted virtually during the participants’ working hours, with each session lasting approximately 90 minutes. The sessions began with brief introductions from the moderator and observer, followed by an introduction of the study aim. Then, each participant introduced herself and described her experiences in diabetes care. The participants were encouraged to engage in open discussion, to build on each other’s points when agreeing or disagreeing and to ask one another follow-up questions. They were also informed that consensus was not necessary on the issues being discussed as all perspectives were of interest and value.

The first author, AD, moderated three of the four FGDs with EB and UFL participating as observers, along with a colleague (EJ) from outside the research team. EB served as the moderator for one FGD, which was conducted without an observer. The moderators’ role was to guide and encourage the discussions to carry on independently relative to set key topics, ask questions to deepen the conversation and ensure that all participants had a chance to share their thoughts and experiences without fear of judgement from the others. The observer took notes on nonverbal cues and followed up with questions at the end of the sessions for the purpose of summarising the discussions (Ivanoff & Hultberg, 2006). All sessions were conducted and video-recorded using a video platform. However, only the audio file was downloaded, saved and transcribed verbatim. Verbatim transcripts were employed for the data analysis.

### Data analysis

The data analysis was conducted in a stepwise manner, following the deliberate and purposeful process outlined by Krueger and Casey (2015) for focus groups, with the study aim guiding the analysis from beginning to end. Because data collection and analysis were conducted concurrently, immediately after each focus-group session, the moderator and observer engaged in a debriefing session to discuss their observations and the key topics addressed.

The audio recordings were repeatedly listened to, and the word-for-word written focus-group transcripts were prepared by AD (Ivanoff & Hultberg, 2006; Krueger & Casey, 2015). The transcripts remained in Swedish throughout the analysis to ensure consistency and preserve the original meaning of the data. All of the authors independently reviewed the transcripts and made detailed notes during the reading process to identify preliminary themes in the data material (Ivanoff & Hultberg, 2006).

AD initially organised the data in the NVivo software programme (version 15.2.2), thereby making it accessible to the entire research team. This process involved identifying and coding relevant segments of transcripts that captured the collective understanding and views of the DSNs related to the study aim. To enhance the analytical depth, the research team—comprising members with diverse academic and professional nursing backgrounds—engaged in repeated discussions and continually reflected on the data throughout the analysis. Codes with similar meanings were systematically organised into preliminary themes guided by patterns of similarity and difference. The collaborative process of interpretation and theme refinement was sustained until a shared interpretation was reached within the research team (Krueger & Casey, 2015), the result being five subthemes and one main theme. To remain close to the raw data, all text was kept in Swedish until the end of the analysis process. All themes were represented in all of the focus groups.

## Results

### Participant characteristics

A total of sixteen DSNs participated in the current study. Homogeneity was ensured by including only female DSNs with shared experience in providing primary diabetes care for patients with T2DM.

Heterogeneity among the participants lay in their age differences, which ranged from 35 to 65 (mean = 50.9, standard deviation [SD] = 8.1) years. In addition, the diversity in terms of their years of experience in the diabetes specialist nursing profession varied from 0.5 to 22 (mean = 8.3, SD = 6.95) years. The variation in terms of their earned higher education credits in diabetes care ranged between 15 and 60 credits (mean = 37.5, SD = 19.75). Further homogeneity was found in terms of whether the DSNs held a master’s degree. Thirteen participants held a master’s degree, six of whom specialised in diabetes care. Moreover, homogeneity was maintained in terms of whether the nurses worked in the public (69%) or private (31%) primary healthcare sector. An overview of the participants’ demographic data is shown in Table I.

**Table I.** Demographic data of the participants in the focus groups (*N* = 16).

Characteristic	Total (*N* = 16)	FGD1 (*n* = 5)	FGD2 (*n* = 4)	FGD3 (*n* = 4)	FGD4 (*n* = 3)
Age, mean (range)	50.9 (35–64)	51.2 (45–60)	50.5 (35–64)	48.5 (39–57)	54.0 (52–56)
Years of experience as a diabetes specialist nurse, mean (range)	8.3 (0.5–22)	7.9 (0.5–22)	8.75 (0.5–16)	4.38 (2–8)	16.0 (1.5–19)
Higher education credits in diabetes care, mean (range)	37.5 (15–60)	39 (15–60)	45 (30–60)	30 (15–60)	35 (15–60)
Master's level degree (%)	13 (81%)	3 (60%)	4 (100%)	3 (75%)	3 (100%)
Working in the public healthcare sector (%)	11 (69%)	4 (80%)	1 (25%)	4 (100%)	2 (67%)
Working in the private healthcare sector (%)	5 (31%)	1 (20%)	3 (75%)	0 (0%)	1 (33%)

### Participants' experiences of professional practice

The analysis resulted in one main theme: “maintaining professionalism in changing and strained primary diabetes healthcare”, which was substantiated by five subthemes presented in Table II. The subthemes that were developed in our analysis were as follows: “requesting managerial prioritising of holistic preventive diabetes care”, “striving for consensus regarding the core concepts that underpin person-centred care”, “desiring allocated time for diabetes care professional tasks”, “balancing between structured protocols and flexible practice” and “confronting the structural prerequisites that influence professional practice”.

**Table II.** The main theme and sub-themes.

Maintaining professionalism in a changing and strained diabetes primary healthcare				
Requesting managerial prioritising of holistic preventive diabetes care	Striving for consensus regarding the core concepts that underpin person-centred care	Desiring allocated time for diabetes care professional tasks	Balancing between structured protocols and flexible practice	Confronting the structural prerequisites that influence professional practice

Our findings are illustrated in the following text, which includes excerpts from the FGDs. To facilitate transparency in the reporting of comments and agreement among participants, each participant is referred to by a unique ID (e.g., P1–P5 and FGD1–4).

### Requesting managerial prioritising of holistic preventive diabetes care

The FGDs revealed that preventive diabetes care varies between healthcare centres and is often influenced by managers’ understanding and support. Long-term preventive diabetes care, a vital part of the work of DSNs, has become less prominent, according to several participants. Instead, they are expected to perform other tasks, such as enhancing digital accessibility. In healthcare units where the manager values long-term efforts to prevent diabetes complications, diabetes care receives high priority in daily practice. In contrast, some units prioritise digital health transformation and accessibility, which can lead to a lower prioritisation of diabetes care.

The DSNs expressed a desire for more precise and consistent guidelines across all primary care units. Current policies were seen as vague and open to interpretation, which led some units with limited managerial understanding of prioritising preventive diabetes care to meet only the minimum standard, such as conducting blood tests once a year. The DSNs emphasised that diabetes care should involve more than just annual blood tests. Across all of the focus groups, the participants consistently expressed that diabetes care requires a holistic approach—one that considers the whole person, not just laboratory results, and they highlighted the value of their preventive work. As one participant in FGD2 illustrated during their discussion:

*P1:* *No, but I’m also thinking—what is our purpose? One of the primary purposes is to prevent diabetes complications, right? And I mean, when blood pressure rises, when blood sugar goes up a bit, and when blood lipids aren’t at reasonable levels, the patient doesn’t feel that. So, we can’t lose them, you know? We need to keep them on the waiting list [and follow up]. And I also feel that if we’re going to have only digital visits, how are we supposed to examine the person’s feet, for example, or check an ECG from time to time—how does that work? This discussion is happening across our entire region now. The healthcare resources are not enough. [Chronic illnesses] are being deprioritised in favour of me sitting in a chat or on the phone.*

The discussion also revealed concerns that some managers lack a comprehensive understanding of preventive diabetes care and the importance of relational continuity and make decisions that overlook the complexity of the needs of patients with chronic conditions when scheduling the DSNs for work tasks that do not require specialised expertise. The DSNs questioned whether managers fully support the implementation of PCC in primary healthcare when these needs are overlooked in everyday work.

In particular, the DSNs highlighted a worrying trend in digital healthcare being imposed from the top down, which has led some healthcare units to transition to only provide digital diabetes care. In those cases, this was experienced as impersonal and fragmented, with patients merely expected to submit blood samples and a digital health declaration form. The DSNs as a group opposed this, noting that some people with diabetes still prefer physical visits a few times a year and use digital services less frequently. They highlighted examples when patients struggled to access 1177, Sweden’s national online healthcare platform, due to limited digital skills, which led to one-way communication where messages were sent from healthcare units without receiving replies from patients. The DSNs emphasised that digital care should supplement, not totally replace, traditional physical diabetes care. While digital care is expected to grow in importance, access to healthcare must remain through both physical and digital in-person consultations, as discussed in FGD4:

*P1: I also think that it [digital care] lies in the future, maybe. Many patients don’t want it [today], but in the future, there may be people who want to measure at home and manage digital care from home. So, I think that will grow.*

*P2: I also think that the future with [glucose sensor] is coming. They [the government] have changed the guidelines for who can have it, and we have also been running it a bit on the sidelines here. I believe this is the future of electronics, as we’ve seen that patients who manage their care with a [sensor] achieve significantly better target results. This, in turn, leads us to the digital side, where we connect with others, and I think there will be even more of that in the future. There will always be a need for physical visits because we cannot fit all into digital formats.*

*P3: I agree with you, but at the same time, we have to push certain aspects to not eliminate all physical visits, specifically for patients who require coming [for physical visits], there should be a possibility to come for a physical visit.*

Furthermore, the DSNs described digital healthcare information as beneficial for patients because it helps them better understand their illnesses and enhance their interactions with healthcare providers to a higher level of knowledge exchange. However, they recognised that the quality of information can vary, and they advise patients to exercise caution and consult them about the information they come across.

### Striving for consensus regarding the core concepts that underpin person-centred care

The DSNs emphasised in all of the focus groups that PCC is a fundamental part of their practice, and they actively incorporate this approach into their daily work and descriptions of how they provide diabetes care. However, not everyone is thoroughly familiar with the core theoretical concepts behind PCC, which indicates it is not yet fully embedded in practice. The DSNs found it challenging to use the concept of partnership when describing their relationships with patients and tended to use alternative wording, as discussed in FGD4:

*P2: I think a partnership for me depends somewhat on how we define it, but I think it is extremely important that you have a care relationship, that you make a plan with the patient, set goals, whether they are interim goals, final goals or varying goals. I may not think that I have a partnership based on my idea of [what a] partnership [entails], but it is absolutely a respectful care interaction where the patient’s needs are prioritised. I mean, I can feel that I have an agenda. However, it must be the patient’s agenda and goals. In the end, I may want to offer some advice on the way, so that the goals are somewhat directed in my direction. However, I am probably not saying a partnership. I would probably say care relationship, then, but we probably mean the same thing. But that word is not right for me.*

*P1: No, it is a term used in person-centred care ethics.*

*P2: Then it’s too modern for me.*

*P3: You can call it a care alliance, that you have an alliance, a relationship with your patient, just like everyone wants to achieve.*

Similarly, the participants in FGD3 discussed the following points:

*P2: But it is quite like that, that the partnership is also part of what we are talking about all the time with person-centred care and so on. I think it’s really important that you get there. You may never get there with everyone, of course. Not everyone is receptive to it or wants it that way, but you have your receptivity, your waiting list, and you see each other year after year. I think that is really important, then it becomes easier for both me and the patient to be honest with each other and have an open dialogue, so I think that, it makes it a lot easier for me, so you come here for a health visit of course and we are going to talk about this and test results and stuff, but I also want to feel that it is pleasant [for them] to come here and it is nice to see each other—“oh, and now we are here again and picking up where we left off last time”. If you still have that relationship with the patient, as I said, I don’t think you end up there with everyone. Of course not, but you try to strive to make the way as easy as possible. And that they feel that you can make contact. I can contact them, and they can contact me, and so on, so that we can, well, easily have a dialogue with each other.*

*P1: But I feel that we already have it in some way with our [diabetes] patients. We have them as we have [by call-recall system], and we have that contact when we meet year after year, and sometimes [we meet] maybe several times a year.*

The DSNs unanimously emphasised that continuity is vital for delivering PCC and fostering collaborative partnerships. Continuity builds trust, boosts collaboration and allows DSNs to explore each patient’s history more thoroughly, thereby providing personalised support and contributing to a balanced power dynamic within the partnership. It also strengthens the perception that partnership is a shared responsibility that requires both parties to take initiative when needed. However, perceived limitations in terms of time and resources within primary healthcare—despite the DSNs undertaking significant, often unseen work—can obstruct the development of trust and continuity and ultimately have a negative effect on the quality of PCC.

### Desiring allocated time for diabetes care professional tasks

The DSNs reported that access to diabetes care varied across primary healthcare units. To improve accessibility for all patients, chronic conditions like diabetes were being deprioritised at some primary healthcare units. Patients with diabetes faced long waiting times for appointments and frequent cancellations by the units, which not only disrupted their continuity of diabetes care but also concerned the DSNs. The DSNs felt this could lead to worse health outcomes in diabetes patients because delays might result in missed opportunities for timely interventions. The DSNs believed that scheduling them for general primary healthcare tasks was an inefficient use of resources and staff and that it also presented an ethical dilemma because they struggled to maintain standards of equitable and person-centred diabetes care.

The DSNs also reported that patients attempted to bypass limited access to them by explicitly requesting to interact with them, even for minor issues that other healthcare staff could manage. Despite busy schedules and being assigned other primary healthcare tasks, the DSNs attempted to accommodate patient requests by fitting them in between their assigned primary healthcare tasks, thereby recognising both the importance patients placed on continuity of care and the trust they had established in the DSNs, as was reflected in FGD4:

*P2: What I often feel is the classic situation, like when you have to call a patient and it’s just, “Oh, I just wanted to renew a prescription for paracetamol”. Yes, maybe my colleague could have handled that, but they’re so attached—they want to do everything with the same person. Now my colleagues have learned to ask many follow-up questions when they talk to them [diabetes patients]. But many times, they say that or come to the reception and say: [Diabetes specialist Nurse] needs to call me.*

*P1: Right.*

*P2: And then they say, “Well, I have diabetes”, and the question isn’t even related to that, but they tie up.*

*P1: Yes, exactly—tying up, that was a good way to put it.*

*P3: Then I think that’s good. It means they trust you. They’re connecting with the person they feel safe with and who can help them with whatever they need.*

Another time-related concern raised by the DSNs was the top-management decision to schedule all annual diabetes visits at a fixed, shorter, centrally determined duration, a practice already implemented in some primary healthcare units. The DSNs described this approach as counterproductive and incompatible with PCC because it predefines the patient’s needs without involving the professionals or the patients themselves in decision-making processes. The DSNs emphasised that no two patient interactions are alike, which makes it impossible to anticipate the primary focus in each session in advance.

The DSNs expressed concerns that shortened annual diabetes visits might limit the opportunities to ask targeted, sensitive questions and could prevent patients from feeling comfortable sharing their personal challenges. This rushed format, the DSNs argued, risked negatively impacting patients’ quality of life and diabetes management. In some units, the DSNs have successfully advocated for retaining individually scheduled visits tailored to the needs of diabetes patients, as discussed in FGD1:

*P1: Now they have announced that the visit should only take half an hour, but I’ve requested an hour and had to ask for it. So we said we have 45 minutes, but I think half an hour—I’m not really sure, it’s hard to say. What do the rest of you think?*

*P4: It’s too short when we see them so rarely [once a year].*

*P1: Yes, I think so too. I don’t understand how it includes so much in that visit. I don’t see how it’s supposed to work.*

*P2: But you know what, I actually think we—we need to set demands. It’s as simple as that.... I didn’t want to agree to having a patient visit limited to 30 minutes, so I went and knocked on the manager’s door and said, kind of like this: You’re very welcome to join me during my annual diabetes visits, but before you do, I want to tell you what it is that I actually do, and then I listed it all. And the one that does it all needs to get the time; simple as that. I can say that she [the manager] never joined my visits and has even expanded our time [for diabetes care].*

*P1: But you [colleague] have 45 minutes, and the visit is supposed to be 30 minutes, and then 15 minutes extra [for documentation]—but it always ends up taking longer, and no one ever sees that.*

*P3: It is incredibly stressful. Although I managed to carry it out, it came at the expense of my own stress and health.*

The DSNs consistently expressed pride in their work and showed strong agreement across all of the focus groups that their professional role is of importance in primary healthcare. They believed their skills and knowledge could be more effectively utilised in primary healthcare. However, some of the DSNs felt that managers did not fully acknowledge their abilities, knowledge and experience, the resources they believed could contribute to improved diabetes care if they were permitted to practice to their full potential. The following reflections were shared by participants during discussions in FGD2 and FGD4:

*P1: I want to emphasise that, organisationally, you always need management on board to provide high-quality care. If management—one— has limited knowledge of the area you work in, two: has stringent economic austerity, then it is going to be hard, and that is the way I perceive it to be currently.*

Another participant shared the following observation.

*P1: Diabetes specialist nurses generally do a fantastic job. If given the opportunities and conditions, we can achieve even more than we do—and relieve the burden on healthcare in many ways. So, I really think you should not skimp on resources there.*

The DSNs collectively emphasised the importance of advocating for improved working conditions by clarifying their influence on decision-makers and reaffirming their dedication to preserving professional autonomy and PCC.

### Balancing between structured protocols and flexible practice

In the discussions, the DSNs emphasised that a diabetes diagnosis, along with emotional and expectation-related experiences, could be perceived as overwhelming for patients, which requires healthcare professionals to be flexible. The DSNs aspired to create opportunities during meetings for patients to share emotions, challenges, concerns and personal goals related to living with diabetes. These conversations sometimes required shifting focus from diabetes management to mental health, family or financial issues, as all of these factors were perceived to influence metabolic control.

The focus group participants unanimously expressed the importance of addressing patients’ immediate concerns during a visit, which the DSNs described as crucial. Without this focus, patients might struggle to concentrate on the initially planned diabetes topics, thereby potentially impeding their ability to meet national diabetes care standards targets. Therefore, the DSNs tailored their approach to their patients’ life circumstances while incorporating national operational requirements. However, they emphasised the importance of demonstrating sensitivity to patients’ situations to ensure success, recognising that each annual visit was unique. In FGD4, the DSNs demonstrated how they subtly incorporated their agenda to meet comparable national targets for good diabetes care without allowing their planned, structured agenda to dominate the meeting.

*P1: I usually start with a conversation about what life is like right now. So that I know a little bit where they are going, I take these tests. However, I usually do it a little while we are talking, and I try to de-dramatise it [the tests]. If everything is good, then everything is good. However, if there is something I feel we need to address, then we will do that. But I always start from where the person is right now [in life, in thoughts], in some way. I try to do that to capture them so they understand the numbers [the test results in relation to their circumstances].*

*P2: I totally agree. I also usually start meetings with [questions]: “How are you now? Do you have any questions?” Because if they [patients] sit there and ache over these things and do not get to say it, we lose focus in our conversation entirely. Then they may have to spout off one or two things that have happened, either in their private lives or something they are dissatisfied with or [if they are] having problems with another illness. And when we have addressed it, as you say, you end up inserting your own agenda at the end.*

This balancing act between emotional support and clinical responsibilities underscores the nuanced role of DSNs. As the DSNs strived to meet both patient needs and a structured agenda, their adaptability was influenced by their level of professional confidence and experience. Experienced DSNs often exercised discretion regarding the topics they addressed during annual visits, occasionally deviated from planned agendas and scheduled additional follow-ups based on patients’ needs. In some cases, a comprehensive diabetes consultation was divided into several sessions because covering multiple key areas in one appointment may be overwhelming for the patient.

In contrast, newly trained DSNs found reassurance in the structured framework of diabetes care, which involved following specific templates to ensure thoroughness in each visit and aligned with organisational and national diabetes targets. They showed limited flexibility and stressed the need for a stricter adherence to templates to ensure patient safety, as discussed in FGD1:

*P1: As a newcomer to this area, I can say that I am quite constrained by a template so that I can include the essential things. I also have to learn this now, so there is less space for [variation]. Of course, you get to know who the patient is and what they want to talk about, but the space is a little less because, yes, I have the template to follow, and I have to do it. Otherwise, I can make mistakes.*

*P2: Yes, I can recognise myself in you. I still consider myself new, even though I have been working for two years. I also have a template I follow to get the things [highlighted] I want out of a conversation. Still, I agree that each patient receives a tailored conversation tailored to the individual in front of us.... You have to put yourself on their level and meet the patient where they are. I would say that how you set it up and convey the information to the person in front of you becomes very individual.*

*P3: You need to break down the information itself, even if it requires two visits, rather than keeping it very compact. Otherwise, they come and do not know, so it is not wrong to take the most important things [at once] and then book another visit. Then it is easier for them to absorb it.*

A shift from strict template adherence to more flexible, experience-based practices demonstrated that the DSNs personalised diabetes care according to individuals’ needs as they gained experience. As the DSNs gained more experience, their professional confidence increased, and they became more skilled at tailoring consultations to align with PCC, relative to patient readiness and responsiveness.

### Confronting the structural prerequisites that influence professional practice

The DSNs consistently described their roles as empathetic, holistic and genuinely person-centred, despite experiencing limited managerial support, the deprioritisation of diabetes care and a demanding work environment. This trend, in which the DSNs’ tasks are deprioritised in some units, stems from the realisation that the rising number of people with diabetes and the strain on the economy require innovative approaches to delivering care. However, they are concerned that the breakdown of diabetes care will ultimately have a negative impact on this patient group and society in the long term, as illustrated by a participant in FGD2, who described it as an emerging natural catastrophe.

*P3: I think this will turn out. In that case, [when] they have abandoned the whole idea of preventing the disease from developing as it is no longer important and prioritised. It will turn out and escalate. Yes, when this tidal wave becomes too large and unmanageable, it will become a tsunami that we cannot handle. In such cases, you need to let it be and accept it as it is.*

The DSNs’ unwavering commitment not only highlighted their professionalism but also underscored their sense of fulfilment and dedication to diabetes care. They found satisfaction in witnessing the positive impact of their efforts on patients’ lives, which was achieved through regular meetings over time, especially when supporting lifestyle interventions. During these meetings, they observed notable improvements in patients’ self-management and health outcomes. This process demands nuanced interpersonal skills to identify the appropriate moments to introduce specific interventions based on a comprehensive understanding of each patient. A key professional skill that was described is knowing when to encourage, coach or hold back, recognising that patients might have other life priorities to manage. Such sensitivity to the timing and life context of each patient highlights the DSNs’ commitment to PCC and their ability to blend clinical expertise with soft, relational skills. A participant in FGD4 further elaborated on the significance of incorporating soft skills and values.

*P2: When I meet the patient, I think, “Okay, who do I have in front of me here?” What kind of person is this? What kind of lifestyle does he have? What are the conditions for improving both one and the other? Are there relatives who need to be contacted?’ A little bit about what is around the patient, rather than just focusing on diabetes, as you also mentioned, X [another participant]. They are usually multi-morbid. What else do we need to consider? And no, I mean, of course, they are in contact with us for their diabetes diagnosis, yes, but it becomes small, I think. You must look at the wholeness. That’s what I mean by soft values.*

The DSNs also described operating at the intersection of medicine and nursing and emphasised the importance of integrating these within diabetes care. However, they relied on their core competency—caring—as the basis of their approach to providing care, and they highlighted aspects of nursing elements in diabetes care, as illustrated in the following FGD3:

*P1: We stand between two worlds where we have the medical part, and that part that is always present when you have diabetes, but we still have nursing, which is our profession, so I still try to lay that as a foundation, that the foundation is always self-care and that is what we should do, so we must not lose that [nursing]. It is essential, so it is still a big part of the visit, absolutely. We cannot dodge that, so we must face it. How far you get in that discussion depends on the person who will receive the information, but that cannot be removed from the visit.*

*P2: Yes, as you say, it is the core of the entire treatment.*

A consensus appeared during FGDs that professional learning and maintaining adequate knowledge were considered essential for the DSNs. However, these aspects were not prioritised in all primary healthcare centres. Several DSNs noted that opportunities for acquiring new knowledge and training during work hours were limited, which was particularly concerning in a rapidly evolving field like diabetes, where continuous professional development is crucial.

This situation also raised questions about professional boundaries. The DSNs emphasised that, like other professional groups, they had a responsibility to maintain their competence through ongoing learning. Nevertheless, this expectation was not always met, as some of the DSNs reported having to update their knowledge outside the workplace context.

## Discussion

The study revealed that DSNs demonstrate a deep professional commitment and pride and find genuine fulfilment in supporting persons with diabetes over time and witnessing their meaningful improvements in self-care and health outcomes. This sense of purpose aligns with the foundational characteristics of a profession, which is defined as a career that requires specialised skills and theoretical knowledge and that is recognised by society for its professional authority and governed by ethical standards (Greenwood, 1957). Nursing, as both a profession and a practice, embodies these attributes through its commitment to equitable, culturally safe and PCC (White et al., 2025).

The DSNs’ reflections illustrated how professional identity is not only shaped through education and training but also reinforced through sustained relationships with patients and the ability to influence long-term health outcomes. Their experiences tell about the distinct culture of nursing grounded in ethical responsibility and specialised expertise. These findings underscore the vital role that DSNs play in primary diabetes care and highlight nursing as a profession that delivers societal value and also fosters personal fulfilment through its unique contributions to patients' health and well-being, in line with previous research (Jutterström et al., 2016; Lawler et al., 2019; Zhu et al., 2024). However, the DSNs in our study voiced concerns that their preventive efforts and professional autonomy were increasingly being compromised by the financial priorities that shape primary healthcare. Their experiences revealed that such structural constraints are narrowing the space for professional actioning, thereby potentially undermining both the quality of care and professional development. Therefore, not prioritising or allocating sufficient time to DSNs to provide high-quality diabetes care and time for work-integrated learning could negatively impact their professional development. This finding aligns with a recent review by Aldahmashi et al. (2024), which showed that nurses in diabetes care play a significant role in healthcare teams by leading interventions to improve patient outcomes. Thus, allocating space for professional actioning and empowering DSNs to take leading positions in diabetes care could promote the quality of diabetes care.

The DSNs in this study intuitively practised PCC by skilfully tailoring lifestyle interventions to each patient’s needs and drawing on advanced interpersonal and clinical judgement to determine when to guide, encourage or respectfully step back. However, not all of the DSNs were familiar with the formal theoretical framework (Ekman et al., 2011), which suggests a gap between practice and conceptual understanding. This disconnect may limit the consistency and depth of PCC implementation and underscore the need for ongoing education and leadership that actively supports and reinforces the theoretical foundations of PCC.

A literature review by Byrne et al. (2020) showed that for nurses to engage in PCC, the organisational environment should be actively supportive and fully reflect PCC principles, which include organisational systems and leadership that prioritise this approach. The experience of the DSNs in our study echoed this finding when they described how top-down decisions in primary healthcare settings directly impact their ability to deliver high-quality diabetes care. A recurring concern among the DSNs was that managerial decisions may lack a nuanced understanding of their professional role and the complexity of their clinical practice, thereby leading to a disconnect that was described in various ways. Some of the DSNs reported feeling pressured to try to maintain the same diabetes care quality despite increasingly constrained working conditions, which led to stress and declining health among DSNs. Others acknowledged that sustaining previous standards was no longer feasible due to reduced time allocations and resource downgrading.

In response, they attempted to shift the responsibility for care prioritisation to their managers, asking for guidance on what aspects of care should be deprioritised or omitted. These accounts reflected the broader systemic tension identified by Byrne et al. (2020), in which institutional demands often conflict with the principles of PCC. Their review highlights the dilemma nurses face between what they *should* do—provide comprehensive, holistic care—and what they *must* do to meet institutional constraints. This tension not only limits their professional autonomy but also restricts their capacity for clinical judgement and decision-making.

Importantly, the DSNs in our study expressed a growing sense of resignation. Despite their professional efforts to resist these changes, they perceived the current trajectory of primary healthcare as unlikely to reverse. Their reflections evoked a powerful metaphor: a “tidal wave” of systemic change that threatens to escalate into a “tsunami”, with far-reaching consequences for both patients and society. This imagery underscores the urgency of addressing structural barriers to PCC and re-evaluating how healthcare systems support the professional practice and well-being of DSNs. These concerns echo the findings from a systematic review by Torrens et al. (2020), which examined barriers to implementing the advanced nurse practitioner role in primary care settings—a role comparable to the Swedish DSN with advanced training in diabetes care. The review identified more than 500 barriers across 54 studies, including limited agreement regarding the level of professional autonomy and the scope of everyday work activities. Additional barriers included challenges in staff management and staffing levels, unclear lines of responsibility, heavy workloads, scheduling difficulties, task characteristics and poor management—many of which were also described by the DSNs in our study.

In this study, the DSNs voiced concerns that some streamlined primary healthcare centres and their managers do not sufficiently support ongoing professional development or fully understand the DSN professional role—both of which are essential for maintaining diabetes-specific competence at the unit. This aligns with Mlambo et al. (2021), whose meta-synthesis highlighted the importance of organisational culture and institutional support for professional growth in ensuring high-quality care and retaining skilled staff. Similarly, Pérez-Francisco et al. (2020) found in their integrative review of primary healthcare that workload affected the health of nurses and decreased the quality of care, while a lower number of errors in patient care was attributed to a higher level of education among nurses.

Morvati et al. (2025) further underscored the value of nurses’ autonomy, learning opportunities and clearly defined roles in fostering a health-promoting work environment. Complementing these findings, a quantitative study on DSNs in Sweden by Dautovic et al. (2025) showed that workplace learning potential was positively associated with work engagement, influence and perceived PCC, while stress and high demands were negatively associated with perceived PCC. Together, the extant research points to the need for organisational prioritisation and manager support for workplace learning to support nurses’ well-being and enable high-quality PCC.

### Strengths and limitations

A strength of FGDs is that they enable participants to interact with each other in discussions on specific topics. An alternative data collection method could have been individual in-depth interviews with DSNs. However, as the study aimed to explore DSNs’ collective experiences of the prerequisites for PCC and their professional practice in diabetes primary healthcare, FGDs were found to be a more suitable research method. As FGDs can help identify group norms and values, encourage open conversation on complex topics, permit expression of criticism that might be left underdeveloped in an individual interview and illuminate the research participants’ perspectives through group debate, Kitzinger (1995) highlighted the importance of FGDs. However, the time available for conducting the focus groups was limited to a 90-minute discussion during the participants’ working hours, which could have been considered constrained. The participants were talkative and wanted to continue the conversation, so the verification provided at the end by the observer was somewhat rushed. Another possible limitation was that one FGD was conducted without an observer, which may have reduced the level of detail in terms of documenting nonverbal interactions and group dynamics. However, the primary data analysed in this study were verbal interactions. Future studies could incorporate both verbal and nonverbal communication.

In the current study, each group comprised three to five participants, which was found to be an adequate group size with an open climate because every participant had time to contribute equally to the discussion and express her experiences. The general focus-group size recommendation varies, and the rule is that the more complex the topic, the more expertise participants have or the stronger their feelings are about it, the more the groups should be restricted to fewer participants (Krueger & Casey, 2015). Different and critical opinions arose during the discussions, which can be seen as a strength because the participants felt comfortable sharing their experiences. Heterogeneity and homogeneity were considered when the group constellations were made. However, most of the participants worked in the public primary healthcare sector. One focus group did not have participants from the private sector, and all of the participants were females, but they differed in terms of work experience in diabetes care and educational background.

## Conclusion

This study highlights both the everyday challenges and the strong commitment of DSNs in delivering person-centred diabetes care within contemporary primary diabetes healthcare. The findings underscore DSNs’ need for organisational support, particularly from managers who value and actively promote PCC, as well as opportunities for continuous professional development. Meeting these needs would better equip DSNs to address the evolving demands of contemporary primary diabetes healthcare. Taken together, such supportive measures could lay the groundwork for a sustainable primary healthcare system for diabetes in the future.

## Data Availability

Due to ethical restrictions, the participants in this study did not agree to share their data publicly. Therefore, supporting data is unavailable.

## Appendix A: Coreq-checklist

Consolidated criteria for reporting qualitative studies (COREQ): a 32-item checklist.

**Table:**

No. item	Guide questions/description		Reported on page #
**Domain 1: Research team and reflexivity**			
***Personal Characteristics***			
1. Interviewer/facilitator	Which author/s conducted the interview or focus group?	The first author, AD, conducted the majority of focus group discussions (*n* = 3) along with the last author, EB (*n* = 1).	5.
2. Credentials	What were the researcher’s credentials? e.g., PhD, MD	The researchers’ credentials are as follows:AD: PhD(c)EB: PhDUFL: PhD	Title page.
3. Occupation	What was their occupation at the time of the study?	The researchers’ occupations are as follows:AD: RN, PhD candidate, lecturer.EB: RN, PhD, senior professor.UFL: RN, MSc, senior lecturer.All have extensive nursing experience.	Title page.
4. Gender	Was the researcher male or female?	All researchers were female gender.	N/A.
5. Experience and training	What experience or training did the researcher have?	EB and UFL are experienced qualitative researchers with numerous publications in the field. AD has completed methodological courses on conducting qualitative focus group research and using NVivo software for data analysis. The research team (AD, UFL, and EB) has previously collaborated on qualitative studies and co-authored related publications.	21.
***Relationship with participants***			
6. Relationship established	Was a relationship established prior to study commencement?	Only for this research.	4.
7. Participant knowledge of the interviewer	What did the participants know about the researcher? e.g., personal goals, reasons for doing the research	The participants were informed about the researcher’s professional background, the purpose of the study, and the researcher's interest in exploring diabetes specialist nurses’ experiences of person-centred care and professional practice.	5.
8. Interviewer characteristics	What characteristics were reported about the interviewer/facilitator? e.g., Bias, assumptions, reasons and interests in the research topic	The focus groups were moderated by a university lecturer and a professor, both with professional expertise in care. To ensure transparency, their backgrounds and motivations for conducting the research were shared with participants. While the moderators were aware of potential biases associated with their professional roles, they took deliberate steps to minimise their influence on the discussions. Data analysis was carried out collaboratively by the entire research team.	5-6.
**Domain 2: study design**			
***Theoretical framework***			
9. Methodological orientation and Theory	What methodological orientation was stated to underpin the study? e.g., grounded theory, discourse analysis, ethnography, phenomenology, content analysis	The focus group discussions and their analysis were guided by Krueger and Casey’s descriptions of focus group methodology	1, 4−6.
***Participant selection***			
10. Sampling	How were participants selected? e.g., purposive, convenience, consecutive, snowball	The participants were purposefully recruited from ten different primary healthcare centres in Western Sweden.	4.
11. Method of approach	How were participants approached? e.g., face-to-face, telephone, mail, email	An invitation to participate was extended to the DSNs, either by phone or email, as recommended after their manager approved, providing both oral and written information about the study. Subsequently, a new contact was made via phone or email with potential participants to address any questions and emphasise the voluntary nature of participation.	4.
12. Sample size	How many participants were in the study?	Sixteen informants working at ten different healthcare centres were included in the study.	4.
13. Non-participation	How many people refused to participate or dropped out? Reasons?	Additionally, three diabetes specialist nurses from two other primary healthcare centres were invited but declined due to heavy workload.	4.
***Setting***			
14. Setting of data collection	Where was the data collected? e.g., home, clinic, workplace	Data was collected via a digital platform.	5.
15. Presence of non-participants	Was anyone else present besides the participants and researchers?	No one else was present in each focus group besides the participants, the moderator, and the observer.	5.
16. Description of sample	What are the important characteristics of the sample? e.g., demographic data, date	Working in primary diabetes care with patients diagnosed with T2DM.	4−5.
***Data collection***			
17. Interview guide	Were questions, prompts, guides provided by the authors? Was it pilot-tested?	The research team developed an interview guide to ensure that key topics—contemporary diabetes care, the role of specialist diabetes nurses, learning, digitalisation, and general health—were consistently addressed across all interviews. The guide included topic areas rather than fixed questions, allowing for flexibility in phrasing. Follow-up questions were used to clarify responses and broaden the scope of the discussions. A pilot interview was not conducted.	6.
18. Repeat interviews	Were repeat interviews carried out? If yes, how many?	No repeat interviews were carried out.	N/A
19. Audio/visual recording	Did the research use audio or visual recording to collect the data?	All sessions were digitally recorded; however, only the audio file was downloaded, saved and transcribed verbatim.	5−6.
20. Field notes	Were field notes made during and/or after the interview or focus group?	Yes, the notes were taken, but only the verbatim transcripts were analysed.	6.
21. Duration	What was the duration of the interviews or focus group?	Each session lasted approximately 90 minutes.	5, 19.
22. Data saturation	Was data saturation discussed?	To enhance analytical depth, the research team—comprising members with diverse academic and professional backgrounds—engaged in reiterated discussions, continually reflecting on the data throughout the analysis.	6.
23. Transcripts returned	Were transcripts returned to participants for comment and/or correction?	The transcripts were not returned to participants for comment or correction.	N/A.
**Domain 3: analysis and findings**			
***Data analysis***			
24. Number of data coders	How many data coders coded the data?	Coding was performed in the software programme NVivo by the first author (AD) in close collaboration with co-authors (EB, UFL).	6, 21.
25. Description of the coding tree	Did authors provide a description of the coding tree?	An explanation of the data analysis procedure, presenting the main team and sub-themes, is provided both in Findings and Table II.	6−7, 24.
26. Derivation of themes	Were themes identified in advance or derived from the data?	The themes deviate from the data. The research team mutually identified higher-order headings of the data content through discussion and interpretation, resulting in one main theme and five sub-themes.	6, 24.
27. Software	What software, if applicable, was used to manage the data?	The software programme NVIVO version 15.2 straightforwardly contained the data and made the analysis process transparent within the research team.	6.
28. Participant checking	Did participants provide feedback on the findings?	No, participants did not provide any feedback on the findings.	N/A
***Reporting***			
29. Quotations presented	Were participant quotations presented to illustrate the themes/findings? Was each quotation identified? e.g., participant number.	Participant quotations are presented in the findings and identified with a specific participant number. Table I shows participant numbers and characteristics.	7−16, 24.
30. Data and findings consistent	Was there consistency between the data presented and the findings?	Yes, there was consistency between the data presented and the findings.	7−16.
31. Clarity of major themes	Were major themes clearly presented in the findings?	Yes, themes were clearly presented in the findings and Table II.	7−16, 24.
32. Clarity of minor themes	Is there a description of diverse cases or discussion of minor themes?	Yes, subthemes are presented in the findings and Table II.	7−16, 24.

^N/A= Not applicable.^ **Developed from:** Tong A, Sainsbury P, Craig J. Consolidated criteria for reporting qualitative research (COREQ): a 32-item checklist for interviews and focus groups. *International Journal for Quality in Health Care*. 2007. Volume 19, Number 6: pp. 349—357.
